# Brazilian airway surgery survey indicates low overall numbers and need for improved teaching skills

**DOI:** 10.1093/icvts/ivad177

**Published:** 2023-11-09

**Authors:** Benoit Jacques Bibas, Helio Minamoto, Paulo Francisco G Cardoso, Mariana Rodrigues Cremonese, Paulo Manuel Pêgo-Fernandes, Ricardo Mingarini Terra

**Affiliations:** Disciplina de Cirurgia Torácica, Instituto do Coração (Incor), Hospital das Clinicas HCFMUSP, Faculdade de Medicina, Universidade de Sao Paulo, Sao Paulo, Brazil; Hospital Israelita Albert Einstein, São Paulo, Brazil; Hospital Municipal Vila Santa Catarina Dr. Gilson de Cássia Marques de Carvalho; Hospital Israelita Albert Einstein, São Paulo, Brazil; Disciplina de Cirurgia Torácica, Instituto do Coração (Incor), Hospital das Clinicas HCFMUSP, Faculdade de Medicina, Universidade de Sao Paulo, Sao Paulo, Brazil; Disciplina de Cirurgia Torácica, Instituto do Coração (Incor), Hospital das Clinicas HCFMUSP, Faculdade de Medicina, Universidade de Sao Paulo, Sao Paulo, Brazil; Disciplina de Cirurgia Torácica, Instituto do Coração (Incor), Hospital das Clinicas HCFMUSP, Faculdade de Medicina, Universidade de Sao Paulo, Sao Paulo, Brazil; Hospital Israelita Albert Einstein, São Paulo, Brazil; Hospital Municipal Vila Santa Catarina Dr. Gilson de Cássia Marques de Carvalho; Hospital Israelita Albert Einstein, São Paulo, Brazil; Disciplina de Cirurgia Torácica, Instituto do Coração (Incor), Hospital das Clinicas HCFMUSP, Faculdade de Medicina, Universidade de Sao Paulo, Sao Paulo, Brazil; Disciplina de Cirurgia Torácica, Instituto do Coração (Incor), Hospital das Clinicas HCFMUSP, Faculdade de Medicina, Universidade de Sao Paulo, Sao Paulo, Brazil; Hospital Israelita Albert Einstein, São Paulo, Brazil

**Keywords:** Airway surgery, Trachea, Tracheal stenosis, Survey, Surgical education, Resident training

## Abstract

**OBJECTIVES:**

The Brazilian Society of Thoracic Surgeons conducted an online survey to determine the number of surgeons that perform adult and paediatric airway surgery and to understand the practice patterns along the country.

**METHODS:**

Active members were electronically invited to complete the questionnaire through the REDCap^R^ platform. Invitations were sent from January to April 2020. The survey encompassed 40 questions that explored 4 different topics in the assessment of tracheal diseases: (i) surgeon’s demography; (ii) institutional profile, (iii) education and training in laryngo-tracheal surgery and (iv) preoperative and postoperative evaluation.

**RESULTS:**

Eighty-nine percentage of the responders declared to perform tracheal surgery with a median of 5 tracheal resection procedures *per* year [interquartile range (IQR) 3–12]. Interaction with other specialties occurs in 37.3% of cases. Access to technology and devices is highly variable across the country. Resident training in airway surgery consists in traditional lectures in 97% of the cases. Training in animals (15.2%), cadavers (12.1%) and simulators (6.1%) are rare. Preoperatory evaluation encompasses flexible bronchoscopy (97.8%) and/or computed tomography (CT) scan of the airways (90.6%). Swallowing (20.1%) and voice (14.4%) disorders are rarely evaluated. Eighty-nine percentage of the surgeons consider bronchoscopy to be the preoperatory gold-standard exam, followed by CT scan (38.8%) and CT-3D reconstruction (37.4%).

**CONCLUSIONS:**

Brazilian surgeons refer that airway resection and reconstruction are part of their current practice, but the total number of procedures per surgeon per year is low. Access to high-end technology and equipment is heterogenous. Training offered to residents in most academic institutions relies on traditional lectures.

**Figure:**
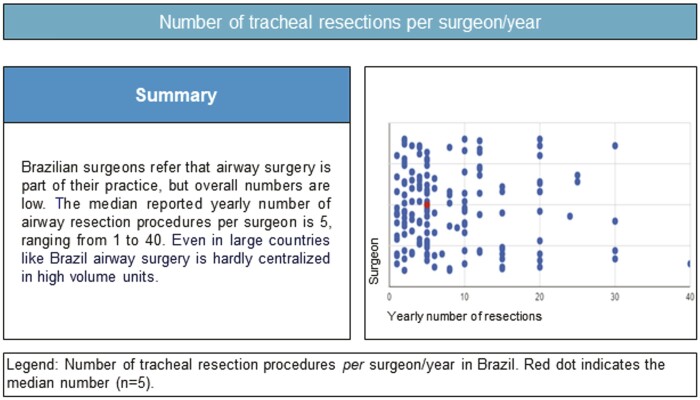

## INTRODUCTION

Surgery of the trachea is a highly specialized field. Due to its complex morphology, anatomy and physiology, this organ represents a special therapeutic challenge [1, 2]. Regardless of the nature of the underlying disorder, good results require a high level of expertise in airway management, a careful diagnosis and interventional planning as well as an experienced surgical team that masters extended operative techniques. Despite improvements in technology such as low cuff pressure and percutaneous dilation tracheostomy approaches, post-intubation tracheal stenosis (PITS) continues to be a major burden, especially in emerging countries [1]. An optimal treatment decision always requires a multidisciplinary assessment of the patient's individual situation by thoracic surgeons, interventional pulmonologists, ear, nose and throat (ENT) surgeons and anaesthesiologists [1, 2].

PITS still represents the most common indication for tracheal resection worldwide. It is a complex problem that often requires multiple interventions for treatment and poses a major burden on the patient’s quality of life, as well as on the health system itself [3, 4]. The reported incidence of PITS varies from 1.8% to 12% of long-term invasively ventilated patients [3]. Most of our patients with PITS are trauma victims of motorcycle accidents, with head injuries that are often submitted to tracheostomy at the primary care centre before they are referred. At present, 80% of our patients with subglottic or tracheal stenosis have a tracheostomy when they are seen at the airway surgery outpatient clinic of the Heart Institute of the University of São Paulo [2–7]. This situation is widespread throughout Brazil and seems to be increasing [2–11]. Nevertheless, the percentage of surgeons that perform airway surgery, where they reside, and the difficulties encountered in their practice are yet unknown.

Therefore, the Brazilian Society of Thoracic Surgeons (BSTS) conducted an online survey in order to determine the number of surgeons that perform adult and paediatric airway surgery, and to understand the distribution of the individuals along the country. Secondary objectives were to determine the practice patterns and difficulties encountered when dealing with tracheal diseases in Brazil.

## MATERIALS AND METHODS

### Ethics statement

This study was approved by the local institutional review board (*Comissão de ética para análise de projetos de pesquisa*-CAPPesq) on 14 August 2019 (IRB number 15364819.2.0000.0065). Formal consent to participate was waived. Participants decided whether to participate in the online survey and were informed that individual responses were to remain anonymous.

We conducted a structured online survey among members of the BSTS. All active members received an e-mail with information about the survey and were invited to complete the questionnaire online through the REDCap^R^ platform. Emails were sent from January to April 2020. The survey was designed by the BSTS Airway Database Committee and encompassed 40 questions that explored 4 different topics in the assessment of tracheal diseases: (i) respondent demographics, (ii) institutional profile, (iii) education and training in laryngo-tracheal surgery and (iv) preoperative and postoperative evaluation (supplementary material). Among BSTS respondents, analyses were limited to those who indicated that their practice involved treating patients with tracheal diseases. The survey was anonymous, and the surgeons could decline to inform their current city of practice, if they wanted to remain fully anonymous (some cities in Brazil have only 1 thoracic surgeon).

Normality analysis was performed with the Kolmogorov–Smirnov test. Categorical variables are presented as absolute numbers and percentage and analysed with Student’s *t*-test or Fisher’s exact test. Continuous variables are presented as mean and standard deviation when they had normal distribution or as median and IQR when they had an asymmetric distribution. The *P*-value was considered statistically significant if <0.05. Stata software, version 13 (StataCorp LP, College Station, TX, USA), was used for the statistical analyses.

## RESULTS

### Respondent demographics

One hundred and ninety-three surgeons from 21 of the 26 states in Brazil responded the survey. The total response rate was 24%. Eighteen responses (9.3%) were excluded due to incomplete data. The mean age of the responders was 45.7 ± 11.6 years (range: 27–73). Thirty-three percentage had over 20 years of clinical practice in thoracic surgery, while 40% had <10 years. Eighty-nine percentage of the surgeons reported that they perform tracheal surgery in their current medical practice. Those that do not will refer patients to other colleagues in public (*n* = 8; 42%) or private (*n* = 9; 47.4%) clinics. Seldomly, patients are referred to other states in Brazil (10.5%). The complete demographic data are depicted in Table 1.

**Table 1:** Respondent demographics

Age (years), median (IQR)	45.7 + 11.6 (27–73)
Gender, *n* (%)	
Male	155 (89.1%)
Female	19 (10.9%)
Current position, *n* (%)	
Resident in thoracic surgery	8 (4.6%)
Staff surgeons	116 (66.7%)
Head/chief of department	50 (28.7%)
Years of medical practice, *n* (%)	
0–5	40 (23%)
5–10	30 (17.2%)
10–15	23 (13.2%)
15–20	23 (13.2%)
>20	58 (33.3%)
Responders *per* state in Brazil	
Acre (*n* = 0)	Alagoas (*n* = 0)
Amapá (*n* = 0)	Amazonas (*n* = 2; 1.3%)
Bahia (*n* = 3; 2%)	Ceará (*n* = 5; 3.3%)
Distrito Federal (*n* = 5; 3.3%)	Espírito Santo (*n* = 3; 2%)
Goiás (*n* = 2; 1.3%)	Maranhão (*n* = 1; 0.7%)
Mato Grosso (*n* = 1; 0.7%)	Mato Grosso do Sul (*n* = 1; 0.7%)
Minas Gerais (*n* = 20; 13.3%)	Pará (*n* = 2; 1.3%)
Paraíba (*n* = 6; 4%)	Paraná (*n* = 12; 8%)
Pernambuco (*n* = 4; 2.7%)	Piauí (*n* = 2; 1.3%)
Rio de Janeiro (*n* = 14; 9.3%)	Rio Grande do Norte (*n* = 2; 1.3%)
Rio Grande do Sul (*n* = 9; 6%)	Roraima (*n* = 0)
Rondônia (*n* = 0)	Santa Catarina (*n* = 8; 5.3%)
São Paulo (*n* = 47; 31.3%)	Sergipe (*n* = 1; 0.7%)
Tocantins (*n* = 0)	

IQR: interquartile range.

**Figure 1:**
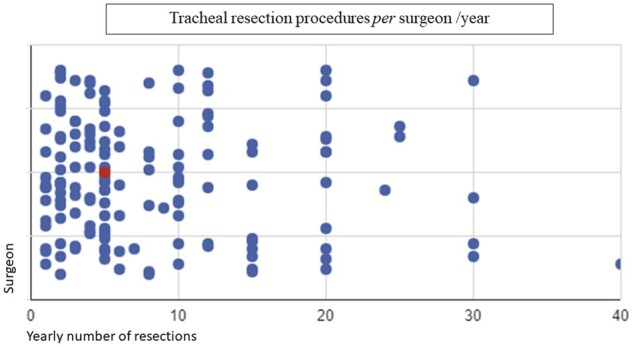
Number of tracheal resection procedures per surgeon/year. Main dot indicates the median value. Median = 5 procedures (interquartile range 3–12).

**Figure 2:**
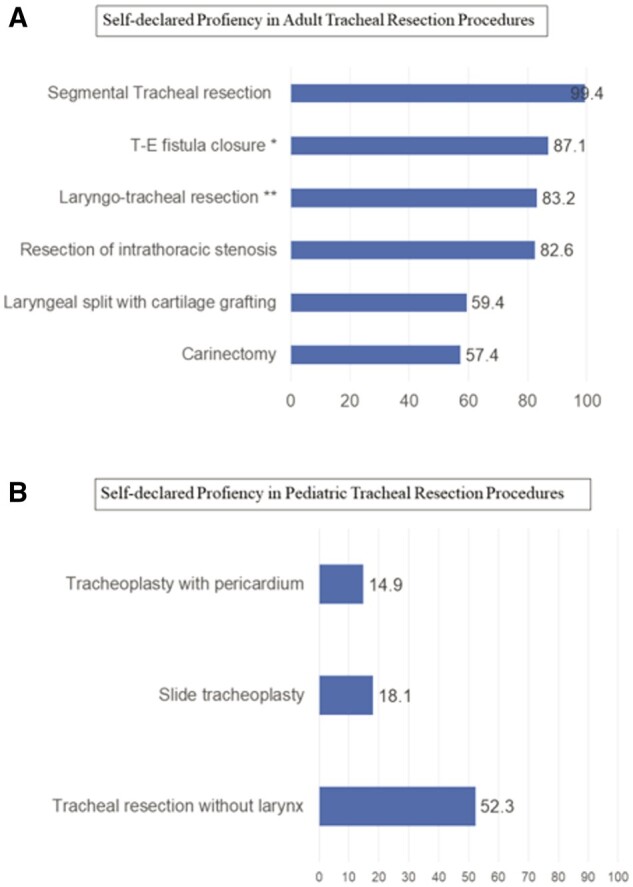
(**A**) Self-declared proficiency in adult tracheal resection procedures. *T-E fistula = tracheo-esophageal fistula. **Laryngo-tracheal resection = Pearson-Grillo procedure. (**B**) Self-declared proficiency in pediatric tracheal resection procedures.

The responders reported a median of 5 tracheal resection procedures per year by each surgeon (IQR 3–12) (Fig. 1). Routine interaction with other specialties, such as head and neck surgeons or ENT surgeons, only occurs in 37.3% of cases. Nonetheless, most surgeons (83.3%) agree that such interactions would probably result in better surgical outcomes. Surgeons were asked to identify which tracheal resection procedures they felt could perform. The result is shown in Fig. 2.

### Institutional profile and training in airway surgery

Sixteen percentage of the surgeons reported that their institutions have a dedicated airway surgery centre/outpatient facility solely dedicated to airway diseases. Furthermore, 32% affirmed that their institution has surgeons who are mainly dedicated to airway surgery. Access to technology and devices was also queried and is depicted in Tables 2 and 3.

**Table 2:** Investigations and adjuncts available to surgeons in their institutions

Adult flexible bronchoscopy	98%
High-resolution CT scan	95.3%
Adult laryngeal mask (various sizes)	94.7%
Adult rigid bronchoscope	84.7%
Paediatric rigid bronchoscope	66%
Paediatric flexible bronchoscope	66%
Videolaryngoscope	58%
Cardiopulmonary bypass	53.3%
High-frequency jet ventilation	36%
ECMO	27.3%

CT: computed tomography; ECMO: extracorporeal membrane oxygenation.

**Table 3:** Availability of silicone stents at the institution

Some sizes available, but not on a regular basis.	43.3%
Stents may be bought individually for each patient, but they are not readily available.	42.7%
Most sizes are available.	13.3%
No access to silicone stents.	0.7%

**Figure 3:**
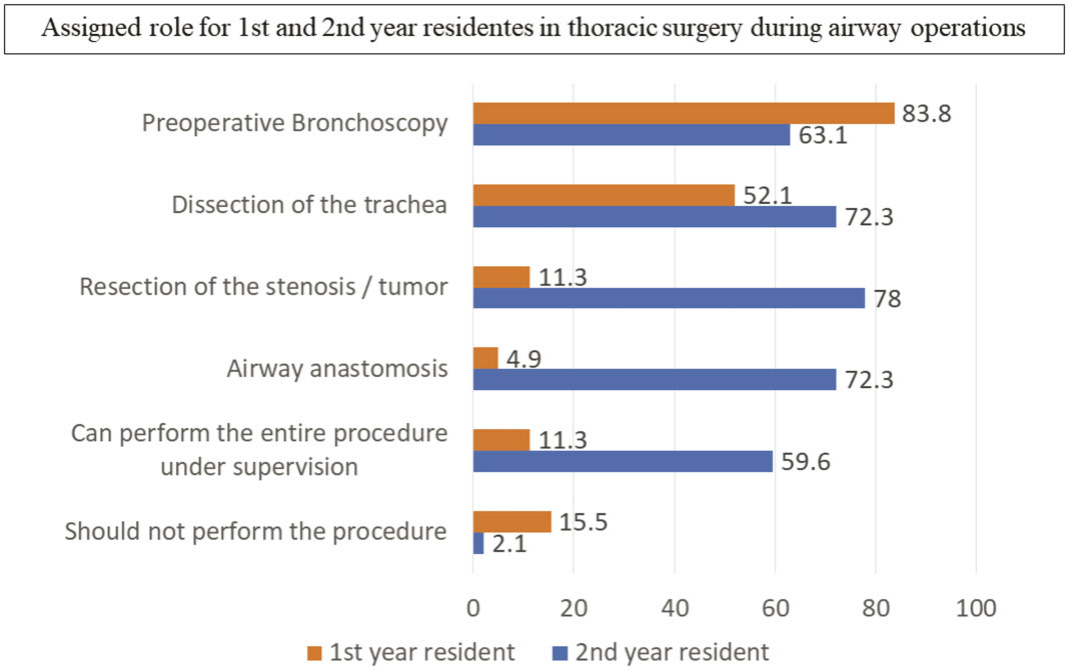
Assigned roles for 1st and 2nd year residents in thoracic surgery during airway operations.

**Figure 4:**
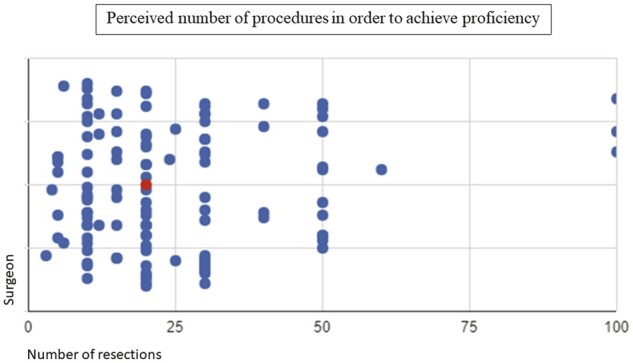
Perceived number of procedures in order to achieve proficiency in airway resection procedures. Main dot indicates the median value. Median = 20 (interquartile range 10–30).

Forty-four percentage of the institutions have residents in thoracic surgery. Fifty-two percentage of the surgeons affirmed that their residents have some training in tracheal surgery. Nonetheless, training consists in traditional lectures in 97% of the cases. Training in animals (15.2%), cadavers (12.1%) and electronic simulators (6.1%) are rare. Nonetheless, most surgeons (86.6%) agree that tracheal surgery is a highly specialized operation and requires a focused and more specific training. The assigned roles for the first- and second-year thoracic surgery resident during the operations are depicted in Fig. 3. Surgeons were asked to respond what would be the number of tracheal resection procedures performed to achieve proficiency. The median response was 20 cases (IQR: 10–30) (Fig. 4). Moreover, 61% of the surgeons affirmed that a tracheal resection performed by a trainee, under supervision, would not have a negative impact in the outcome of the operation.

### Preoperative and postoperative evaluation

**Figure 5:**
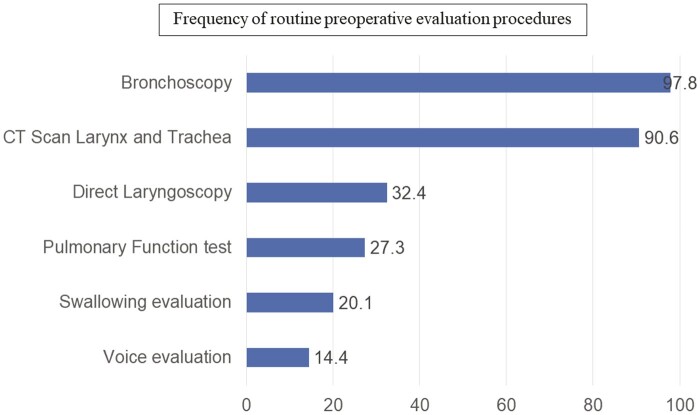
Frequency of routine preoperative evaluation procedures.

Preoperatory evaluation is done mainly with flexible bronchoscopy (97.8%) and/or computed tomography (CT) scan of the larynx and trachea (90.6%) (Fig. 5). Swallowing (20.1%) and voice (14.4%) disorders are rarely evaluated prior to the operation. Eighty-nine percentage of the surgeons consider bronchoscopy to be the best exam to determine the type of operation to be performed, followed by CT scan (38.8%) and CT-3D reconstruction (37.4%).

Postoperative care is still widely performed at the intensive care unit (79.6%). Routine bronchoscopy to evaluate the anastomosis was a question of debate. Forty-three percentage perform routine endoscopic examination of the anastomosis. However, 52.6% will perform a bronchoscopy only if signs of complications are present, such as subcutaneous emphysema or deep wound infection.

## DISCUSSION

In the study presented herein, we aimed to analyse how airway surgery is performed and taught throughout Brazil. The online survey was distributed by the BSTS mail-list and encompassed key areas that we thought needed clarification: (i) who currently performs airway surgery in Brazil, (ii) institutional profiles, (iii) what is the status of education and training in laryngo-tracheal surgery and (iv) preoperative and postoperative evaluation. Those queries are strategic in order to plan improvement in education, improve access to health treatment and reduce inequity throughout the country.

Even though tracheal resection seems to play a part in the Brazilian thoracic surgeon routine, the overall yearly number of airway resections per surgeon is low. Also, due to memory bias, this number could be even smaller. Furthermore, the survey is a fraction of the totality of thoracic surgeons in Brazil, and those who responded are probably the ones who are more involved in airway surgery. It is also of great concern that most surgeons believe that 20 airway resections would be sufficient to reach proficiency, since high-volume airway centres will typically operate on 20–30 cases each year [7, 12–14]. A similar situation seems to occur in North America. A recent study performed an analysis of tracheal surgical outcomes by using The Society of Thoracic Surgeons General Thoracic Surgery Database. Over 1500 cases from 107 centres were identified. Nine centres performed >4 procedures/year and accounted for 50% of the total volume [15]. The majority of centres performed <4 cases *per* year.

This leads to the issue of the learning curve in airway surgery. To the best of our knowledge, no studies evaluated the learning curve in airway resection procedures, but this knowledge is well-stablished in minimally invasive anatomical lung resection procedures [16, 17]. The learning curve has always an initial learning period, followed by a consolidation phase and a final period, in which the technique is mastered. Some series report that it usually takes between 100 and 200 cases to fully master a technique [16, 17]. However, due to the low volume of airway resections, such numbers are quite difficult, if not impossible, to achieve at a low-middle volume centre. Thus, the creation of national referral centres with dedicated airway surgeons along the country could, in due time, improve outcomes and facilitate the training of residents and senior trainees. This high level of expertise has been demonstrated in a recent manuscript by Slama *et al.* [18]. In carefully selected patients treated in a specialized centre, tracheal or laryngotracheal resection after previous tracheal interventions provides comparable outcome to primary surgery. This may represent a change in paradigm since tracheal reoperations have historically been associated with poorer outcomes [3, 7, 14].

Our results indicate that routine interaction with other specialties, such as head and neck surgeons or ENT surgeons, only occurs in a third of the cases, despite the fact that most thoracic surgeons (83.3%) believe that such cooperation would lead to better surgical outcomes. Having multidisciplinary conferences can be challenging, but online meetings have gained widespread use during the COVID-19 pandemic and can facilitate discussions. Ideally, complex airway stenosis cases should be addressed in ‘airways boards’, in the same manner that oncologic cases are managed in tumour boards. In fact, there is solid evidence that a multidisciplinary board is highly beneficial, as it allows physicians to deal rapidly with simple cases on a systematic basis, to give more attention to the most complicated situations and to offer every patient the benefit of a multidisciplinary approach [19].

Moreover, multidisciplinary management could widen the scope of surgical outcomes after airway resection and include evaluation of functional parameters. Our results indicate that voice and swallowing disorders are seldomly investigated in Brazil. Nevertheless, voice alterations without recurrent nerve palsy are somewhat common after laryngotracheal resection and are a serious handicap. This aspect is underexposed in current literature and deserves further attention during preoperative counselling and patient follow-up [20].

Another aspect that the survey brought to light is that the training offered to residents in most academic institutions is somewhat outdated. With growing work-time restrictions and public expectations, the Halstedian educational model of ‘see one, do one, teach one’ is unfit for the modern raining of thoracic residents [21]. Hoetzenecker *et al.* [22] demonstrated that 3D models of complex glotto-subglottic airway stenosis are a supplementary tool in the preoperative assessment of patients with PITS. They may help residents and surgeons less familiar with airway surgery to understand the complex 3D anatomy and the relationship of a scar stenosis to functionally important structures of the laryngotracheal region [23]. Such reconstructions are simple and might overcome the downsides of traditional two-dimensional CT images or endoscopy exams.

Our data also shows that simulators for airway reconstruction training are rarely used in Brazil. Thus, trainees learn in real-time operations, with little prior exposure to airway anatomy and reconstruction techniques. However, studies have shown that simulators could be useful for developing laryngeal and airway surgery skills [23, 24]. Simulators can be high-fidelity or low-fidelity and can be extremely inexpensive while still effective. Simulators for laryngeal injections, bronchoscopy, intubation, flexible laryngoscopy, cricothyroidotomy, tracheostomy and airway reconstruction are available for obtaining and maintaining these skills [23, 24].

In the recent years, the BSTS and other academic institutions have tried to provide a standardized curriculum for thoracic surgery residents and trainees in Brazil. Training consists of an extensive online course that encompasses the main topics in thoracic surgery, with professors and mentors from several institutions. The course includes lectures, chat forums with case-discussions, pro-con debates, and hands-on activities (twice a year). Airway surgery and respiratory endoscopy is part of this curriculum, but we feel that dedicated training is required. Since most centres have low airway surgery volume, it is of utmost importance that young surgeons be trained in airway resection techniques in animal models.

Access to technology was also a major concern, and results show that there is great disparity in the availability of high-end technology and exams. This is most evident when we analyse advance methods of cardiopulmonary support and ventilation. Most institutions do not have access to ECMO, cardiopulmonary bypass or even high-frequency jet ventilation. This support has become the backbone for complex airway resections, especially in paediatric airway surgery [25]. Furthermore, only 66% of the institutions have a full set of paediatric airway equipment. This too reflects the low level of expertise in paediatric airway surgery demonstrated in the survey. As a matter of fact, only 18% of the responders considered themselves able to perform a slide tracheoplasty, and 50% a simple tracheal resection without laryngeal involvement.

### Limitations

Our study has several limitations. First, our survey had a 24% response rate, which is low, but not bad for online surveys. Nonetheless, it reflects a fraction of the total population. Moreover, we had to exclude 9.3% of the responses due to incomplete data. Strategies to increase the response rate such as personal contact, poll at national meetings and links through online messaging apps could have been used. Another issue that could have influenced the results is memory bias. As some responses relied on surgical numbers, it is likely that the numbers are somewhat estimates, rather than high-fidelity data. On the other hand, the survey was not mandatory, and only surgeons that routinely perform airway surgery participated, which may have reduced the variability of our sample.

## CONCLUSION

In conclusion, the survey conducted by the BSTS revealed an interesting paradigm in Brazil. Many thoracic surgeons refer that airway resection and reconstruction is a part of their current practice. Nevertheless, total numbers per surgeon are quite low. Access to high-end technology and equipment for the paediatric population is heterogenous. Training offered to residents in most academic institutions still relies on traditional lectures. Newer technologies such as 3D reconstruction models and high-fidelity simulators could be included.

## Supplementary Material

ivad177_Supplementary_Data

## Data Availability

The data contained in this article refer to the answers provided by the responders from the Brazilian Society of Thoracic Surgery and are currently allocated inside the RedCap^R^ platform. All relevant data are within the article and its Supporting Information files. The data underlying this article may be shared on reasonable request to the corresponding author. **Benoit Jacques Bibas:** Conceptualization; Data curation; Formal analysis; Investigation; Methodology; Project administration; Writing—original draft; Writing—review & editing. **Helio Minamoto:** Methodology; Supervision; Writing—original draft; Writing—review & editing. **Paulo Francisco G. Cardoso:** Formal analysis; Investigation; Methodology; Writing—review & editing. **Mariana Rodrigues Cremonese:** Writing—review & editing. **Paulo Manuel Pêgo-Fernandes:** Methodology; Supervision; Writing—review & editing. **Ricardo Mingarini Terra:** Formal analysis; Methodology; Supervision; Writing—review & editing. Interdisciplinary CardioVascular and Thoracic Surgery thanks Madhuri Rao, Nabil Hussein, Clemens Aigner and the other, anonymous reviewer(s) for their contribution to the peer review process of this article.

## Notes

Presented as poster at the 30th ESTS Meeting, The Hague, Netherlands, 19–21 June 2022.

## ABBREVIATIONS

BSTS: Brazilian Society of Thoracic Surgeons
CT: Computed tomography
ENT: Ear, nose and throat
IQR: Interquartile range
PITS: Post-intubation tracheal stenosis

## References

[1] Bibas BJ , Peitl-Gregorio PH, Cremonese MR, Terra RM. Tracheobronchial surgery in emerging countries. Thorac Surg Clin 2022;32:373–81. 35961745 10.1016/j.thorsurg.2022.04.004

[2] Bibas BJ , Cardoso PFG, Salati M, Minamoto H, Luiz Tamagno MF, Terra RM et al Health-related quality of life evaluation in patients with non-surgical benign tracheal stenosis. J Thorac Dis 2018;10:4782–8. 30233850 10.21037/jtd.2018.07.80 PMC6129892

[3] Cardoso PFG , Bibas BJ, Minamoto H, Pêgo-Fernandes PM. Prophylaxis and treatment of complications after tracheal resection. Thorac Surg Clin 2018;28:227–41. 29627057 10.1016/j.thorsurg.2018.01.008

[4] Bibas BJ , Cardoso PFG, Hoetzenecker K. The burden of tracheal stenosis and tracheal diseases health-care costs in the 21^st^ century. Transl Cancer Res 2020;9:2095–6. 35117562 10.21037/tcr.2020.02.59 PMC8798798

[5] Bianchi ET , Guerreiro Cardoso PF, Minamoto H, Bibas BJ, Salati M, Pego-Fernandes PM; Surgery of the Digestive Tract Group. Impact of fundoplication for gastroesophageal reflux in the outcome of benign tracheal stenosis. J Thorac Cardiovasc Surg 2019;158:1698–706. 31587887 10.1016/j.jtcvs.2019.07.111

[6] Bibas BJ , Guerreiro Cardoso PF, Minamoto H, Eloy-Pereira LP, Tamagno MF, Terra RM et al Surgical management of benign acquired tracheoesophageal fistulas: a ten-year experience. Ann Thorac Surg 2016;102:1081–7. 27329192 10.1016/j.athoracsur.2016.04.029

[7] Bibas BJ , Terra RM, Oliveira Junior AL, Tamagno MF, Minamoto H, Cardoso PF et al Predictors for postoperative complications after tracheal resection. Ann Thorac Surg 2014;98:277–82. 24820396 10.1016/j.athoracsur.2014.03.019

[8] Gaspar MTDC , Maximiano LF, Minamoto H, Otoch JP. Tracheal stenosis due to endotracheal tube cuff hyperinflation: a preventable complication. Autops Case Rep 2019;9:e2018072. 30863738 10.4322/acr.2018.072 PMC6394355

[9] Maunsell R , Lacerda NS, Prata L, Brandão M. Pediatric airway reconstruction: results after implementation of an airway team in Brazil. Braz J Otorhinolaryngol 2020;86:157–64. 30583942 10.1016/j.bjorl.2018.10.011 PMC9422558

[10] Camargo JJ , Machuca TN, Camargo SM, Lobato VF, Medina CR. Surgical treatment of benign tracheo-oesophageal fistulas with tracheal resection and oesophageal primary closure: is the muscle flap really necessary? Eur J Cardiothorac Surg 2010;37:576–80. 19800809 10.1016/j.ejcts.2009.08.023

[11] Rodrigues Cremonese M , Bibas BJ, Minamoto H, Pêgo-Fernandes PM, Guerreiro Cardoso PF. Hybrid desobstruction of blind-end subglottic stenosis with long-term stenting. Ann Thorac Surg 2021;112:e393–e395. 33753061 10.1016/j.athoracsur.2021.03.027

[12] Schweiger T , Roesner I, de Faria Soares Rodrigues I, Evermann M, Frick AE, Denk-Linnert DM et al Functional outcome after single-stage laryngotracheal reconstruction with rib cartilage grafting. J Thorac Cardiovasc Surg 2022;163:313–22.e3. 33640122 10.1016/j.jtcvs.2020.11.155

[13] Maurizi G , Vanni C, Rendina EA, Ciccone AM, Ibrahim M, Andreetti C et al Surgery for laryngotracheal stenosis: improved results. J Thorac Cardiovasc Surg 2021;161:845–52. 33451851 10.1016/j.jtcvs.2020.12.023

[14] Wright CD , Grillo HC, Wain JC, Wong DR, Donahue DM, Gaissert HA et al Anastomotic complications after tracheal resection: prognostic factors and management. J Thorac Cardiovasc Surg 2004;128:731–9. doi: 10.1016/j.jtcvs.2004.07.005. PMID: 15514601. 15514601

[15] Stanifer BP , Andrei AC, Liu M, Meyerson SL, Bharat A, Odell DD et al Short-term outcomes of tracheal resection in the Society of Thoracic Surgeons database. Ann Thorac Surg 2018;106:1612–8. 30240762 10.1016/j.athoracsur.2018.07.041 PMC6696934

[16] Vieira A , Bourdages-Pageau E, Kennedy K, Ugalde PA. The learning curve on uniportal video-assisted thoracic surgery: an analysis of proficiency. J Thorac Cardiovasc Surg 2020;159:2487–95.e2. 31926696 10.1016/j.jtcvs.2019.11.006

[17] Zhang Y , Liu S, Han Y, Xiang J, Cerfolio RJ, Li H. Robotic anatomical segmentectomy: an analysis of the learning curve. Ann Thorac Surg 2019;107:1515–22. 30578780 10.1016/j.athoracsur.2018.11.041

[18] Slama A , Zaatar M, Demir M, Okumus O, Mattheis S, Kampe S et al Tracheal resection after previous treatment provides comparable outcome to primary surgery. Thorac Cardiovasc Surg 2022;70:505–12. 34062598 10.1055/s-0041-1728772

[19] Riquet M , Mordant P, Henni M, Wermert D, Fabre-Guillevin E, Cazes A et al Should all cases of lung cancer be presented at Tumor Board Conferences? Thorac Surg Clin 2013;23:123–8. 23566964 10.1016/j.thorsurg.2013.01.003

[20] Timman ST , Schoemaker C, Li WWL, Marres HAM, Honings J, Morshuis WJ et al Functional outcome after (laryngo)tracheal resection and reconstruction for acquired benign (laryngo)tracheal stenosis. Ann Cardiothorac Surg 2018;7:227–36. 29707500 10.21037/acs.2018.03.07 PMC5900076

[21] Nashaat A , Sidhu HS, Yatham S, Al-Azzawi M, Preece R. Simulation training for lobectomy: a review of current literature and future directions. Eur J Cardiothorac Surg 2019;55:386–94. 30137279 10.1093/ejcts/ezy276

[22] Hoetzenecker K , Chan HHL, Frommlet F, Schweiger T, Keshavjee S, Waddell TK et al 3D models in the diagnosis of subglottic airway stenosis. Ann Thorac Surg 2019;107:1860–5. 30825452 10.1016/j.athoracsur.2019.01.045

[23] Reighard CL , Green K, Powell AR, Rooney DM, Zopf DA. Development of a high-fidelity subglottic stenosis simulator for laryngotracheal reconstruction rehearsal using 3D printing. Int J Pediatr Otorhinolaryngol 2019;124:134–8. 31195305 10.1016/j.ijporl.2019.05.027

[24] Burns JA , Adkins LK, Dailey S, Klein AM. Simulators for Laryngeal and Airway Surgery. Otolaryngol Clin North Am 2017;50:903–22. 28669461 10.1016/j.otc.2017.05.003

[25] Pola Dos Reis F , Minamoto H, Bibas BJ, Minamoto FEN, Cardoso PFG, Caneo LF et al Treatment of tracheal stenosis with extracorporeal membrane oxygenation support in infants and newborns. Artif Organs 2021;45:748–53. 33350476 10.1111/aor.13898

