# Supplementary material for: Brazilian airway surgery survey indicates low overall numbers and need for improved teaching skills
Source: Interdiscip Cardiovasc Thorac Surg. 2023 Nov 9;38(2):ivad177. doi: 10.1093/icvts/ivad177 (PMC10858343; doi:10.1093/icvts/ivad177)
Supplement: ivad177_Supplementary_Data [file ivad177_supplementary_data.zip › Questionario VA SBCT_english version.pdf]

## Patterns of Care in Laryngo-Tracheal Surgery

**Demography**

1. Age:
Free text (years)

2. Sex:
 Male
 Female

3. Current Position:
 Resident in Thoracic Surgery
 Staff Surgeon – Thoracic Surgery
 Head of Thoracic Surgery Department

4. Years of practice in thoracic surgery (after finishing residency)
 0-5 years
 5-10 years
 10-15 years
 15-20 years
 > 20 years

5. Do you consider that you are qualified to perform tracheal, and / or laryngotracheal resection operations?
 Yes
 No

6. If not, where do you send patients to?
 University Service (same State/region)
 Private Service (same State/region)
 Another colleague with more experience (same state/region)
 Reference center of another State / country (public or private)

7. If yes, what type of operation do you consider yourself able to perform?
 In adults - Tracheal Resection with tracheo-tracheal anastomosis (without laryngeal involvement)
 In adults - Resection of the trachea and anterior portion of the cricoid cartilage arch (Subglottic Resection - Pearson-Grillo Procedure)
 In adults - Anterior and posterior laryngofissure with costal cartilage graft, or other procedures of larynx widening with cartilage graft.
 In adults - Carinectomy with reconstruction
 In adults - Correction of benign tracheoesophageal fistula.
 In children - Correction of tracheal stenosis with tracheo-tracheal anastomosis
 In children - Slide tracheoplasty
 In children - Tracheoplasty with pericardium

Service Features
8. What city do you work in?
Free text

9. In what state do you work
List of States

10. Where you perform Laryngotracheal Surgery (multiple choice)
 Public Hospital
 Private Hospital
 Philanthropic Institution
 Public University Hospital
 Private University Hospital

11. Is there interaction between medical specialties for joint operations? (*ENT surgeons, head and neck surgeons)*
 Yes
 No
 Eventually

12. Do you think that the interaction between these specialties would result in a better postoperative result?
 Yes
 No
 Not necessarily

13. What equipment do you have in your hospital: (multiple choice)
 Adult flexible bronchoscope
 Pediatric flexible bronchoscope
 Adult rigid bronchoscope
 Pediatric rigid bronchoscope
 High Resolution Tomography
 Adult laryngeal mask (various sizes)
 Pediatric laryngeal mask (various sizes)
 Videolaryngoscope
 Other rigid or semi-rigid intubation devices
 ECMO
 Extra Extra-corporeal circulation
 Jet ventilation

14. What is the availability of silicone stents at your institution:
 I don`t have them
 I do not have routinely, but it is possible to get through specific purchase orders.
 I have some types of stents, but not all sizes and types I would like to
 I have all types and sizes I need

15. In the event of stent placement, who performs the procedure?
 The Thoracic Surgeon
 Endoscopist // Bronchoscopist // Interventional Pulmonologist
 Procedure is performed together (Surgeon + Bronchoscopist/ Interventional Pulmonologist)

16. How many tracheal or laryngotracheal operations (resections) do you perform per year?
- 0-5
- 5-10
- 10-15
- 15-20
- > 20

17. How many tracheal or laryngotracheal operations (resections) are performed at your institution each year?
- 0-5
- 5-10
- 10-15
- 15-20
- > 20

18. Do you consider that there is a number of operations necessary to acquire proficiency in laryngo-tracheal surgery?
- Yes
- No

19. If yes, what is the number: free text

**Education and Training**

20. Is there an outpatient clinic in your institution dedicated to tracheal diseases?
- Yes
- No

21. Is there in your institution a group of surgeons dedicated mainly to tracheal diseases?
- Yes
- No

22. Do you believe that laryngotracheal surgery requires specific training, different than general thoracic surgery?
- Yes
- No

23. Does the institution where you work have residents of thoracic surgery?
- Yes
- No

24. If yes, do residents have specific training in tracheal surgery?
- Yes
- No

25. If yes ---- What training?
- Theoretical classes
- Simulators
- Training in corpses

□ Animal training

26. Do you think it would be important for residents of Thoracic Surgery to have training focused on tracheal diseases?
□ Yes
□ No

27. During operations, what is the function (in general terms) of a 1st year resident in laryngo-tracheal surgery? (multiple choice)
□ Perform preoperative Bronchoscopy
□ Tracheal dissection only
□ Stenosis / tumor resection
□ Perform the anastomosis
□ They can perform the entire procedure, provided they have previously performed similar operations.
□ A resident must not perform this type of operation

28. During operations, what is the function (in general terms) of the 2nd year resident in a laryngo-tracheal surgery? (multiple choice)
□ Perform preoperative Bronchoscopy
□ Tracheal dissection only
□ Stenosis / tumor resection
□ Perform the anastomosis
□ They can perform the entire procedure, provided they have previously performed similar operations.
□ A resident must not perform this type of operation

29. Do you consider that a tracheal or laryngotracheal resection performed by a resident physician could have a negative impact on the outcome of the case, even if it is performed under supervision?
□ Yes
□ No

**Pre and Post-Operative Evaluation**

30. What examinations are performed routinely in the preoperative evaluation of a tracheal resection? (without laryngeal involvement)
□ CT Scan (Larynx and Trachea)
□ Flexible or rigid bronchoscopy
□ Direct laryngoscopy
□ Pulmonary function test
□ Specific voice assessment
□ Deglutition evaluation
□ others ___free text

31. What examinations are performed routinely in the preoperative evaluation of a laryngotracheal resection? (with laryngeal involvement)
□ Laryngeal and Tracheal Tomography
□ Flexible or rigid bronchoscopy
□ Direct laryngoscopy
□ Pulmonary function test

□ Specific voice assessment
□ Deglutition evaluation
□ others ___free text

32. Which preoperative examination best defines the type of operation to be performed? (multiple choice)
□ Bronchoscopy
□ Tomography (axial, coronal and sagittal sections)
□ Tomography with 3D reconstruction

33. At what moment the preoperative bronchoscopy is performed?
□ I don´t do it routinely
□ At the outpatient facility
□ In the operating room, just before the procedure.

34. Who performs the bronchoscopy?
□ Endoscopist
□ The Surgeon who will perform the resection procedure

35. Is there any specific voice evaluation performed in the preoperative period?
□ Yes -- free text
□ No

36. Is there any specific swallowing evaluation performed in the preoperative period?
□ Yes -- free text
□ No

37. Do you perform a routine bronchoscopy with the patient still in hospital after tracheal or laryngotracheal resection?
□ *Yes*
□ No
□ Only if there are signs of complication (surgical wound infection, subcutaneous emphysema, etc.)

**Follow-up PO**

38. How is the postoperative surgical outcome evaluated? (multiple choice)
□ Clinical evaluation of symptoms
□ Routine bronchoscopy
□ Pulmonary Function Test
□ CT Scan
□ Other_____free text

39. Is there any specific voice evaluation in the postoperative period?
□ Yes ___free text
□ No

40. Is there any specific swallowing evaluation in the postoperative period?
□ Yes ___free text
□ No
